# Comparison of T Tube Ileostomy and Bishop Koop Ileostomy for the Management of Uncomplicated Meconium Ileus

**DOI:** 10.21699/jns.v6i3.617

**Published:** 2017-08-10

**Authors:** Md Samiul Hasan, Ashrarur Rahman Mitul, Sabbir Karim, Kazi Md Noor-ul Ferdous, M Kabirul Islam

**Affiliations:** Department of Surgery, Dhaka Shishu (Children) Hospital, Dhaka

**Keywords:** Meconium ileus, T-tube ileostomy, Bishop Koop ileostomy

## Abstract

Background: Meconium ileus is a common cause of neonatal intestinal obstruction. Various surgical procedures are in practice for uncomplicated meconium ileus. Bishop Koop ileostomy allows distal passage of gut content and uses the distal absorptive area. T tube ileostomy avoids the need for gut resection and formal closure of stoma. The aim of this prospective interventional study was to compare the outcome of T-tube ileostomy and Bishop Koop ileostomy for the treatment of uncomplicated meconium ileus.

Materials and methods: It was a prospective interventional study from January 2015 to December 2016. Patients were randomly assigned to the T-tube ileostomy group (group A) and Bishop Koop ileostomy group (group B). The patients were followed up for 6 weeks post-operatively. Surgical outcomes between the two groups were compared.

Results: The age range of the patients was 1 to 7 days; majority of the patients were males. Mean operation time of group A (60.76±5.81 minutes) and group B (87.05±6.49 minutes) showed significant difference (p =0.0001). After operation, mean time to start bowel movements in group A (4.90±1.41days) and group B (6.53±2.58 days) showed significant difference (p= 0.020). Times to establish oral feeding, irrigation tube removal and postoperative complications showed no significant difference. All patients that survived in the group B required formal stoma closure, while in the group A stomas closed spontaneously. One patient in the group A had intraperitoneal leakage leading to mortality after second operation. Four patients had leakage in the group B; 2 of them died.

Conclusions: T-tube ileostomy was found as an effective and safe procedure for the management of uncomplicated meconium ileus.

## INTRODUCTION

Meconium ileus is a common cause of neonatal intestinal obstruction [1]. Here obstruction occur secondary to the intraluminal accumulation of inspissated and desiccated meconium [1]. About 50% patients present with complications like volvulus, atresia, perforation and meconium cyst [1]. In the past, meconium ileus was considered to be closely associated with cystic fibrosis (CF). However, recent studies demonstrate that meconium ileus occurs frequently in the absence of CF as well [2]. Uncomplicated meconium ileus can be treated with therapeutic contrast enemas as described by Noblett [3]. Several complications have been reported following Gastrografin enema like perforation, necrotizing enterocolitis, shock and occasional death [4]. Therefore, Noblett set some criteria to be fulfilled before performing this procedure [3]. Copeland et al. also reported diminishing role of the contrast enema [4].

Options for surgical management of uncomplicated meconium ileus include resection of dilated ileum along with a Bishop–Koop ileostomy, Santulli procedure or Mikulicz procedure. These are extensive operations associated with reduction of length of the gut and high stoma output. A second surgery to close the stoma is also mandatory [1]. Bishop Koop ileostomy has been in use in our center for many years as it allows distal passage of gut content and uses the distal absorptive area. With times, normal bowel movement establishes and stoma becomes nonfunctioning. It is an extensive operative procedure, include resection of dilated gut and end to side anastomosis. Incidence of postoperative complication is higher and a second surgery to close the stoma is required [1]. In contrast, T-tube ileostomy includes enterotomy, evacuation of thick meconium and placement of T-tube for postoperative irrigation. Tube enterostomy without gut resection was first performed by O’Neill which was modified by Harberg et al. using T tube [5]. T-tube ileostomy has several advantages. This procedure does not require gut resection and there is no intraperitoneal anastomosis. After extraction of T-tube, the wound heal spontaneously and second operation to close the stoma is not required [6]. The aim of this study was to compare the outcome of T-tube ileostomy and Bishop Koop ileostomy for the treatment of uncomplicated meconium ileus.

## MATERIALS AND METHODS

This was a hospital-based prospective interventional study conducted on the patients with uncomplicated meconium ileus presenting to the Division of Pediatric Surgery, Bangladesh Institute of Child Health & Dhaka Shishu (Children) Hospital, Dhaka from January 2015 to December 2016. Exclusion criteria included preterm, low birth weight neonates and neonates with other congenital anomalies. A convenience sample size of 42 patients was chosen; these 42 patients were randomly divided in two equal groups (Group A – T tube ileostomy and Group B – Bishop Koop ileostomy). Ethical permission was taken from hospital ethical committee. Data were collected in a pre-designed, semi-structured questionnaire, after taking consent from guardians in the consent form.

Operative procedure

After initial resuscitation, all patients underwent laparotomy under general anaesthesia. The abdomen was opened through right supra umbilical transverse incision. The diagnosis was confirmed at laparotomy by presence of meconium pellets causing ileal obstruction.

T tube ileostomy:

After confirmation of diagnosis at laparotomy, an enterotomy was made on antimesenteric border of ileum, 3-4 cm proximal to distal narrow segment. Thick meconium and pellets were evacuated through the enterotomy with minimal bowel handling. A 14/16 Fr T-tube was inserted through the enterotomy and was secured with double purse string suture with catgut. Irrigation was given with saline and 5% N-acetyl cysteine solution. The T-tube was brought out through a stab incision in the right iliac fossa and enterostomy was secured to the anterior abdominal wall.

Bishop Koop ileostomy:

After confirmation of diagnosis at laparotomy, the segment of the maximally distended hypertrophied ileum was resected. After manual decompression of the distal gut, the end of the proximal gut was anastomosed to the side of the distal gut approximately 4 cm from the end. The distal limb was then brought out through a stab wound in the right iliac fossa. A 10Fr feeding tube was kept in distal limb for postoperative irrigation with N-acetyl cysteine solution.

Postoperatively from 1st POD, tube irrigation with 10 ml 2% N-acetyl cysteine 12 hourly was performed in both the groups; the end point was establishment of spontaneous bowel movement. This was followed by initiation of oral feeds.

In case of absent bowel movement, the irrigation was continued up to 10th POD, provided there were no signs of peritonitis and patient’s vital signs were within normal limit. If there were signs of peritonitis or no bowel movement beyond 10th POD, re-laparotomy was considered.

All the patients were advised to attend in the OPD on 2nd, 4th and 6th week after operation. If stoma did not close spontaneously even after 6 weeks, the parents were advised for hospital admission for formal closure of stoma.

The statistical analysis was conducted using SPSS (statistical package for social science) version 20 statistical software. Associations of continuous data were assessed using student t- test. Associations of categorical data were assessed using Chi-square test and Fisher’s exact test. For both test, p<0.05 was considered significant.

## RESULTS

A total of 42 patients were included, 21 each in both the groups. These patients were well matched as respect of age, sex and weight (Table1).

**Figure:**
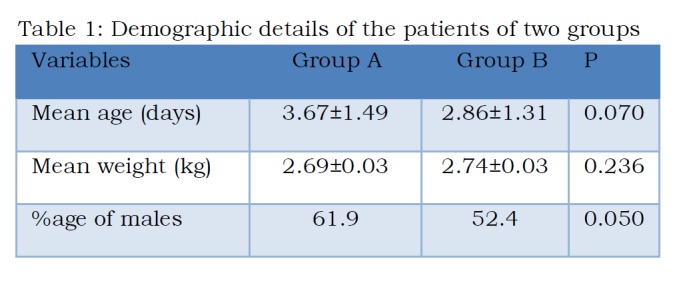
Table 1: Demographic details of the patients of two groups

The mean operation times in groups A and B were 60.76±5.81 minutes and 87.05±6.49 minutes respectively. The difference was statistically significant (p<0.0001) (Table 2).

**Figure:**
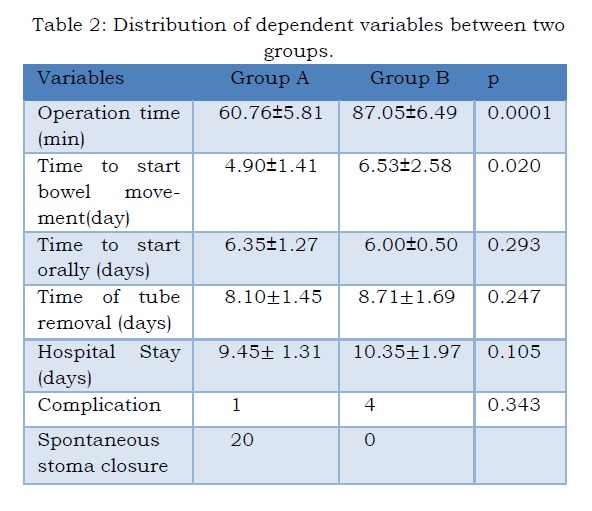
Table 2: Distribution of dependent variables between two groups

Mean times of establishment of bowel movements postoperatively in groups A and B were 4.90±1.41days and 6.53±2.58 days respectively; these were statistically significantly different (p=0.020). One patient in the group A had intraperitoneal leakage leading to mortality after second operation. Four patients had leakage in the group B; 2 of them died of sepsis.

The mean times to oral feeding after surgery in groups A and B were 6.35±1.27 days and 6.00±0.50 days respectively, which were not statistically significantly different (p=0.293). The mean times taken before irrigation tubes could be removed in groups A and B were 8.10±1.45 days and 8.10±1.45 days respectively, which were not statistically significantly different (p=0.247). The mean durations of hospital stay in were in groups A and B were 9.45±1.31days10.35±1.97 days respectively, which were not statistically significantly different (p=0.105).

In group A, most of the neonates (95.24%, n=20) had no post-operative complications. In this group, only 1 neonate had intraperitoneal leakage. On the other hand, in group B, 81.0% (n=17) neonates had no post-operative complications. In this group, 4 neonates (19.0%) had intraperitoneal leakage. This result showed no statistical difference between two groups as the p=0.343.

In group A, almost all of the neonates (n=20, 95.2%) survived after the operative procedure. On the other hand, in group B, 90.5% (n=19) neonates survived after the operative procedure.

Spontaneous closure of stoma occurred in all 20 patients of group A, but no spontaneous stoma closure occurred in group B.

## DISCUSSION

The mean operation time was significantly shorter in T tube ileostomy. Bhattacharyaya et al. had supported T tube ileostomy citing less time taken as compared to the other methods [7]. However, in our study the surgeries were performed by three different surgeons and the operating times could have differed had a single surgeon performed all surgeries.

Tube irrigation was given with 2% N-acetyl cysteine 2 times daily from 1st post-operative day. Bhattacharyaya et al. also used 2% N-acetyl cysteine [7], whereas Mak et al used N-acetyl cysteine in 50% cases and pancreatic enzyme in 50% cases for tube irrigation [8]. Venugopal and Shandling [9] used 5% N acetyl cysteine for irrigation.

Time to start bowel movement was significantly shorter in T tube group. Bhattacharyaya et al. reported bowel movement on approximately 7th (range 5th to 9th) postoperative day [7] and Mak et al reported it on 5th (range 2 -12 days) day [8] in case of T tube ileostomy. These finding was almost similar with this study. In Bishop-Koop ileostomy, as the dilated gut is resected, it is expected to establish bowel movement early. But in comparison to T tube ileostomy, it took more time. It may be due to more tissue handling and presence of proximal stoma in Bishop Koop ileostomy.

Timing of oral feeding, tube removal and hospital stay in T tube group matched with Harberg et al., Bhattacharyaya et al and Mak et al. and [5,7,8] and there was no significant difference between two groups.

In our study, one neonate had intraperitoneal leakage after T tube ileostomy and died after second laparotomy. No complication was reported by Millar et al. [6] and Bhattacharyaya et al. [7] Mak et al. had reported persistent obstruction in 3 patients out of 23 with T-tube irrigation [8]. In Bishop Koop ileostomy, four neonates had intraperitoneal leakage, which subsequently caused sepsis; two of these had died after second laparotomy. Millar et al. reported 1 case of intraperitoneal leakage & death and 4 cases of ileostomy site sepsis out of 10 patients who underwent Bishop Koop ileostomy [6]. Wit et al. had one case of anastomotic leakage out of 27 patients with Bishop Koop ileostomy [10].

Overall survival rate was 92.9% in the present study. This result matches with the study of Rescorla where survival at 1 year was 92% in patients with uncomplicated meconium ileus [11].

Recently, Ziegler had quoted 95-100% survival of neonates with meconium ileus and had accredited it to optimum surgical, pulmonary and nutritional care [1]. Preterm, low birth weight neonates and neonates with other congenital anomalies were excluded from our study, which would have negatively influenced the morbidity and mortality.

Spontaneous closure of stoma occurred in all patients survived with T-tube ileostomy, but formal stoma closure was required in all survived patients with Bishop Koop ileostomy. This finding is similar with the study of Millar et al. [6]. Bhattacharyaya et al. and Mak et al. also reported spontaneous closure of stoma after T tube removal. [7,8]. This avoids a second laparotomy to close stoma in a compromised child. Nguyen et al. also reported formal closure of stoma in all 9 patients after Bishop Koop ileostomy [12].

The study has some limitations. Operations were not performed by a single surgeon, which might have an influence on the result. The study was conducted in Dhaka Shishu (Children) Hospital, which may not be a true reflection of the whole country. Cystic fibrosis was not excluded in study population. Due to short study period, small sample size was taken and longtime observation could not be performed which may influence the external and internal validity of the study.

## CONCLUSION

This study concludes that T-tube ileostomy is an effective and safe procedure for the management of uncomplicated meconium ileus. It requires less operation time and is associated with less complication in comparison with Bishop Koop ileostomy. The stomas in T-tube ileostomy close spontaneously and do not require formal closure under general anesthesia, which is inevitable in Bishop Koop ileostomy.

## Footnotes

**Source of Support:** None

**Conflict of Interest:** None
